# 16S rRNA analysis of diversity of manure microbial community in dairy farm environment

**DOI:** 10.1371/journal.pone.0190126

**Published:** 2018-01-05

**Authors:** Pramod Pandey, Colleen Chiu, Max Miao, Yi Wang, Matthew Settles, Noelia Silva del Rio, Alejandro Castillo, Alex Souza, Richard Pereira, Richard Jeannotte

**Affiliations:** 1 Department of Population Health and Reproduction, School of Veterinary Medicine, University of California, Davis, California, United States of America; 2 Department of Plant Sciences, College of Agricultural and Environmental Sciences, University of California, Davis, California, United States of America; 3 Department of Plant Pathology, University of Wisconsin, Madison, Wisconsin, United States of America; 4 Department of Biological and Agricultural Engineering, University of California, Davis, California, United States of America; 5 Genome Center Bioinformatics Core, University of California, Davis, California, United States of America; 6 University of California Cooperative Extension, Veterinary Medicine Teaching and Research Center, Tulare, California, United States of America; 7 University of California Cooperative Extension, Merced, California, United States of America; 8 University of California Cooperative Extension, Tulare, California, United States of America; 9 Universidad de Tarapacá, Arica, Chile; University of Oklahoma, UNITED STATES

## Abstract

Dairy farms generate a considerable amount of manure, which is applied in cropland as fertilizer. While the use of manure as fertilizer reduces the application of chemical fertilizers, the main concern with regards to manure application is microbial pollution. Manure is a reservoir of a broad range of microbial populations, including pathogens, which have potential to cause contamination and pose risks to public and animal health. Despite the widespread use of manure fertilizer, the change in microbial diversity of manure under various treatment processes is still not well-understood. We hypothesize that the microbial population of animal waste changes with manure handling used in a farm environment. Consequential microbial risk caused by animal manure may depend on manure handling. In this study, a reconnaissance effort for sampling dairy manure in California Central Valley followed by 16S rRNA analysis of content and diversity was undertaken to understand the microbiome of manure after various handling processes. The microbial community analysis of manure revealed that the population in liquid manure differs from that in solid manure. For instance, the bacteria of genus *Sulfuriomonas* were unique in liquid samples, while the bacteria of genus *Thermos* were observed only in solid samples. Bacteria of genus *Clostridium* were present in both solid and liquid samples. The population among liquid samples was comparable, as was the population among solid samples. These findings suggest that the mode of manure application (i.e., liquid versus solid) could have a potential impact on the microbiome of cropland receiving manure as fertilizers.

## 1. Introduction

It has been reported that bacteria loads associated with enormous amount of animal waste produced in the U.S. are the leading cause of impairment for rivers and streams [1, 2, 3]. However, the impact of animal waste-borne microbiomes on environment including soil, water, and plant is not well-understood. The United State Department of Agriculture (USDA) estimates that there are approximately 450,000 animal feeding operations (AFOs) in the U.S., which include beef cattle, dairy, poultry and swine production industries. Annually, over 2 billion tons of animal manure are generated in the U.S. In California alone, 60 million tons (@30 kg/head) of manure are produced annually by 5.2 million cattle and calves, and a considerable portion of the manure is applied onto cropland as fertilizers [4, 5]. While the use of manure as fertilizer in cropland has numerous benefits, such as reducing chemical fertilizer application, additional understanding of how animal waste-borne microbiomes could impact cropland and public health is needed to utilize the full potential of manure and to understand any consequential negative impacts of manure on cropland and environment [6, 7, 8].

Elevated pathogen/pathogen indicator levels in surface and ground water and their potential linkages with animal waste have received considerable public attention because of associated public and animal health risks and produce contamination [9, 10]. In general, the use of fresh and untreated manure as fertilizers has a greater potential to increase pathogen loads in cropland, and subsequently these bacterial populations can be transported to rivers and streams during rainfall/runoff events [11, 12, 13]. Furthermore, the use of untreated manure as fertilizer can facilitate the transfer of harmful bacterial population to ready-to-eat crop [8, 14]. To control the bacterial loads in manure used as fertilizer, several manure treatment practices such as composting, anaerobic lagoon systems, and anaerobic digestions are used [15]. Previous studies showed that pathogens such as *E*. *coli*, *Salmonella*, and *Listeria* in dairy manure are reduced through the application of these waste treatment processes [16, 17, 18], though the complete elimination of these pathogens by these processes is uncertain. Further, the existing knowledge is weak in terms of changes in microbial community in manure after various manure handling processes, such as solid-liquid separation, manure piling, and storage lagoon.

**Fig 1:**
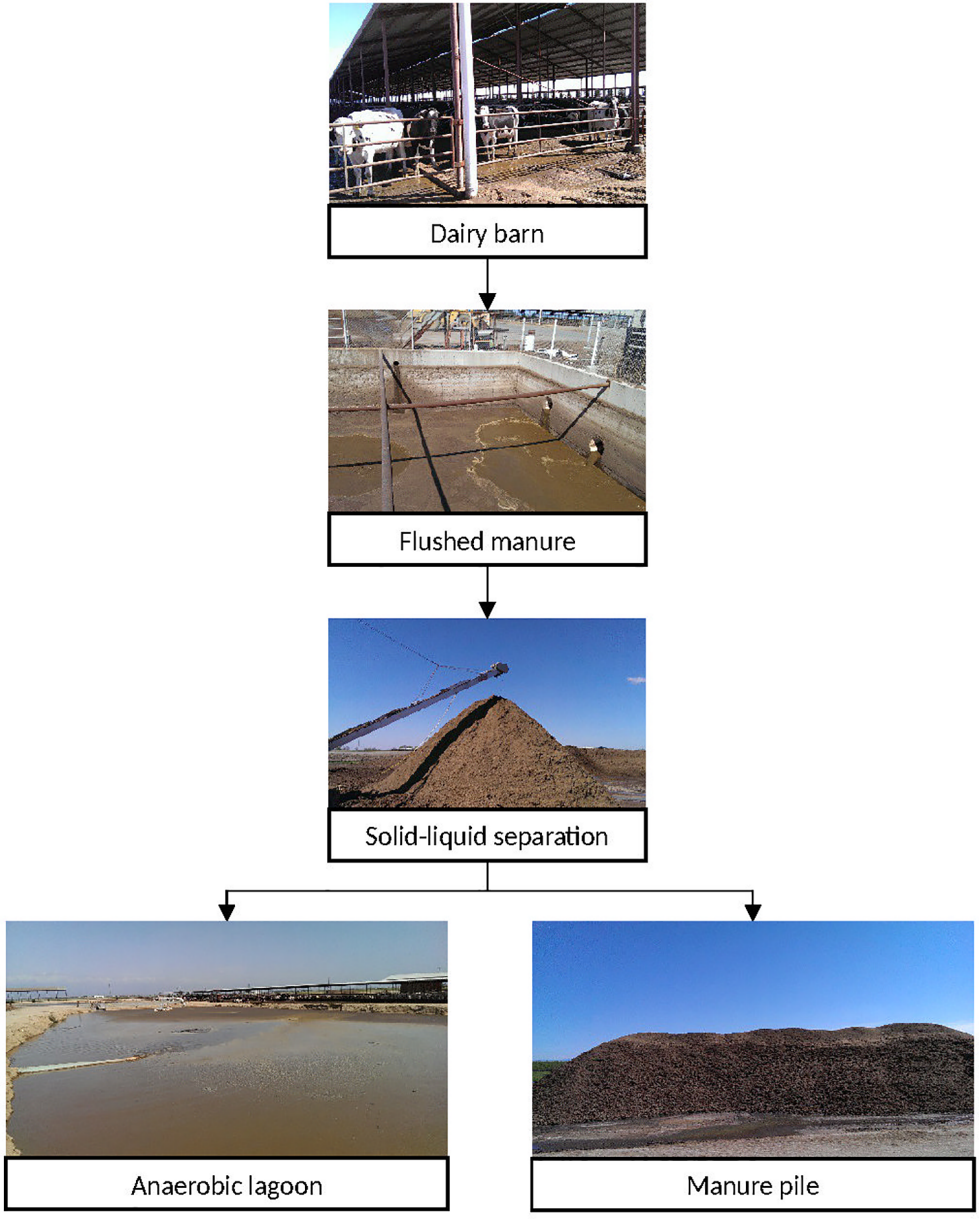
Typical elements of flushed manure handling in dairy farms in Central Valley California, USA.

In a typical, large California dairy, both liquid and solid manure are produced by flush manure management systems, which are common in California’s Central Valley. In such systems, a dairy barn is flushed with water, and flushed manure is passed through solid-manure separation systems, where liquid manure is separated from solid (Fig 1). Solid streams are stored in the form of piles, and liquid manure streams are stored in lagoon systems prior to the application of manure into the cropland as fertilizers [2]. Although both lagoon systems and compost piles are used extensively to manage dairy manure in California, the efficacies and effectiveness of these manure handling processes for regulating microbial population are not well-understood.

For these practical reasons that have considerable impact on agriculture, manure management in dairy farms, and its application in cropland, we hypothesized that microbial quality of dairy manure should change with on-farm manure handling/treatment processes. Moreover, this change in microbial population should be consistent from one farm to another. Such changes or shifts in microbial population of manure—and continuous use of that manure as fertilizer in a cropland for long period—have potential to impact the microbiome of cropland receiving manure as fertilizer. Therefore, the understanding of how the dominant bacterial community levels changes in typical dairy manure management practices in a farm environment is essential.

Although numerous previous studies targeted investigation of the inactivation of selective bacterial pathogens such as *E*. *coli*, *Salmonella*, *Listeria* under specific conditions [15, 19, 20], these studies mainly focused on understanding of selective human pathogen inactivation in various treatment processes. The insights of how various microbial populations at genus level change in particular processes are crucial, however, not well-reported. Further, having such information can help improve the currently-available manure management techniques, and support decision-making in terms of using manure as fertilizer in a specific cropland.

Previous studies have used high-throughput microbial community profiling methods to gain insights into microbial community distribution in different environments [21, 22, 23]. Amplicon-based community analysis has been used to determine the microbial communities in various samples, including food samples, anaerobic sludge, biosolids, natural environments, and agricultural grasslands [24, 25, 26, 27]. These methods have also been applied in raw dairy manure [28, 29, 30, 31, 32, 33]. However, the application of these methods to understand the microbial communities of manure fertilizers processed at various levels of treatment has not been explored yet.

As a test of our hypothesis to determine the differences in microbiome of manure fertilizers, the goal of this study was to quantify the microbial population levels of various forms of dairy manure, such as liquid and solid produced in a typical dairy farm environment in California Central Valley. The objectives of the study are to: 1) determine the dominant microbial communities in solid and liquid forms of dairy manure fertilizer; and 2) understand the changes in microbiome of manure after solid-liquid separation, lagoon storage, and manure piling (commonly used manure-handling processes in a dairy farm). We anticipate that the outcomes of this study will reveal greater understanding in terms of microbial quality of dairy manure fertilizers, and will help in making informed decisions. Further, improved insights will help in advancing dairy manure management, manure application, and understanding the environmental and public health risks associated with animal waste-borne microbial pathogens.

## 2. Materials and methods

### 2.1 Solid and liquid manure sampling

**Fig 2:**
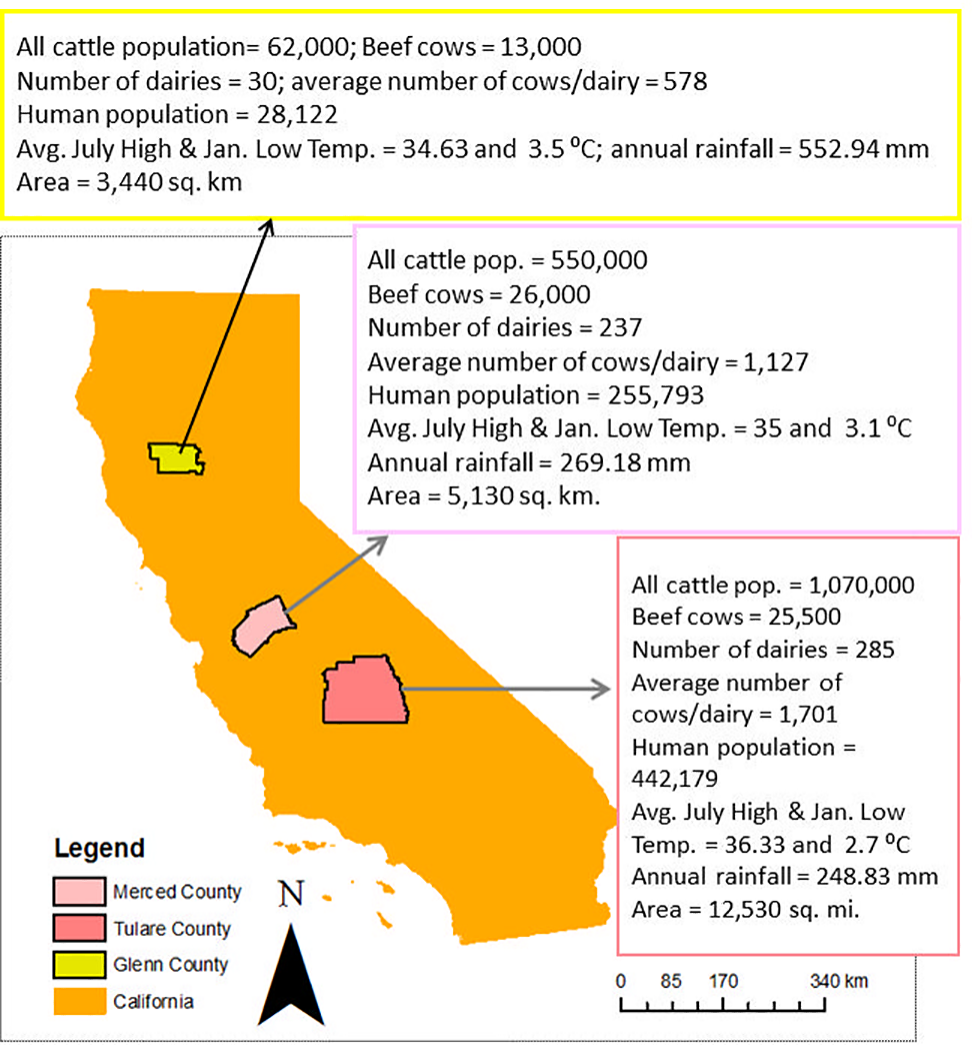
Descriptions of dairy facilities in Merced, Tulare, Glenn counties.

The solid and liquid samples in dairy farms were collected from California Central Valley, which has the most densely populated dairy farms in California. Fig 2 shows county maps of Tulare, Glenn, and Merced, including the herd size in Tulare and Merced Counties. For the current study, we collected 33 manure samples, which include solid and liquid manure. Solid manure samples were collected from manure piles (0–6 months old) located in dairy facilities, while liquid samples were collected from liquid manure storage ponds (primary and secondary lagoons) (0–6 months old storage) as well as from flushed manure pits.

Dairy facilities used for sample collections are located in three counties (Merced, Tulare, and Glenn) (the identities of specific locations are kept anonymous). From each dairy facility, we collected one liter of liquid manure sample in sterile bottles from each pond, and 600 g of solid manure in sterile bottles from each pile. Immediately after collection, samples were transported using a cooler (≈4°C) and subsequently stored at -20°C prior to analysis. For analysis, samples were thawed at room temperature. The liquid manure samples collected from flushed pits were termed as Flush Manure (FM). The solid samples collected from piles that were less than 2 weeks old were termed as Fresh Pile (FP), while older piles were termed as Compost Pile (CP). It is important to note that the studied CP does not necessarily mean the sample was subjected to standard composting processes, where maintaining the thermophilic temperature and mixing is necessary. The liquid manure samples collected from Primary Lagoons and Secondary Lagoons were termed as PL and SL, respectively.

### 2.2 Molecular analyses of microbial communities

In solid samples, 0.25 g of sample was processed by the MO BIO PowerSoil® kit. In sludge-like liquid samples, 10 mL of sample were centrifuged in 50 mL Falcon tubes at 10,000 × g for 10 minutes at room temperature to obtain pellets before bead beating. In liquid samples with clear-to-low turbidity, 10–200 mL of sample were filtered through a Millipore filter (0.45-μm pore size), which was inserted into a 5 mL collection tube and processed by the MO BIO PowerWater® isolation kit. The purification process for both kits involves homogenizing the environmental samples in step-wise, specially formulated solutions for cell lysis, inhibitor removal, DNA binding and DNA elution. Extracted DNA samples were diluted to 5 ng/μL and stored at -20°C prior to PCR amplifications.

We used high-throughput sequencing methods for characterizing the variation in taxonomic marker gene sequences for understanding the microbial diversity of dairy manure samples in farm environment. For microbial community analysis, we sequenced the V3 and V4 hypervariable regions of the 16S rRNA gene, using 16S Amplicon PCR Forward Primer = 5’ (TCGTCGGCAGCGTCAGATGTGTATAAGAGACAGCCTACGGGNGGCWGCAG) and 16S Amplicon PCR Reverse Primer = 5’ (GTCTCGTGGGCTCGGAGATGTGTATAAGAGACAGGACTACHVGGGTATCTAATCC). In the first round of PCR, the template specific primers extracted the region of interest, and in the second PCR, sequencing adapters and barcodes were attached to the sequences. The second round PCR primers included the suitable Illumina adapters with the reverse primers. PCR products were pooled together in equimolar concentrations for sequencing.

Multiplexing of samples was facilitated using an error-correcting 12-bp barcode unique to each sample. During the PCR indexing, dual indices and Illumina sequencing adapters were attached to the amplicons using the Illumina Nextera^®^ XT Index Kit. Verification of attachment by monitoring base pair size was done using the Agilent^®^ Bioanalyzer DNA 1000. The quality of the amplicons was checked by DNA gel electrophoresis, using the DNA extracts as the control. All amplicons were normalized by dilution to the lowest concentrated sample (6.33 ng/uL) and pooled in equimolar amounts, which was calculated using the formula from SureSelect Strand-Specific mRNA Library Preparation for Illumina^®^ Platform Sequencing [Volume of Index = (final desired volume of pool × final desired concentration of all DNA in the pool) / (number of samples × initial concentration of each indexed sample)]. The quality and concentration of the pooled library were determined using the Agilent^®^ Bioanalyzer High Sensitivity DNA 1000. All sequencing runs were conducted in the DNA Technologies Core Facility of the Genome Center at the University of California-Davis. During sequencing, raw DNA reads from an Illumina^®^ MiSeq platform were assigned to samples multiplexed and classified using the previous approach [34, 35]. The custom python application dbcAmplicons (https://github.com/msettles/dbcAmplicons) was used to identify and assign reads by both expected barcode and primer sequences. A python function convert2ReadsTo4Reads was used to extract dual barcodes information associated with each read into a four read fastq set. For quality filtering, barcodes and primers were allowed to have 1 and 4 mismatch, respectively. Subsequently, sequence reads were trimmed of their primer sequence and merged into a single amplicon sequence using FLASH2 [34]. Assignment of sequence to phylotypes was performed using the RDP Bayesian classifier. The RDP Bayesian Classifier determines a confidence value by bootstrapping, and it provides a confidence score for each level of the classification. The confidence score of 50% or higher is recommended for taxonomic classification [36, 37]. In this study, the phylotype taxonomy was determined using the RDP classifier with a bootstrap confidence score of 50% or greater [35, 38, 39]. Further, the relative abundance of different bacterial taxa in each sample was used as covariates in stepwise discriminant analysis models built in JMP Pro 13.0. In the discriminant analysis used in our study, taxa were removed in a stepwise manner until only variables with a P value <0.05 were retained in the final model. Canonical scores for these analyses were used to create graphical display of the results for taxon in the analyses. A canonical cut-off loading value of ± 0.3 was used [40, 41].

## 3. Results

### 3.1 The microbial communities of manure fertilizer post manure handling

The microbial diversity assessment of solid (CP and FP) and liquid wastes (FM, PL, and SL) using phylotype taxonomy resulted in a total taxa of 1818. Approximately 85% of 1818 taxa were classified at the genera level and 10% at the family level. In FP solid samples, sequence reads varied from 13,950 to 453,625 with an average of 153,316. In solid samples from CP pile, sequence reads varied from 15,798 to 1,092,032 with an average of 296,153. The average reads for FM were 333,450 with range from 5,242 to 989,040. In PL and SL liquid samples, the average sequence reads were 186,341 (varied from 39,481 to 372,734) and 130,888 (varied from 18,982 to 348,441), respectively.

**Fig 3:**
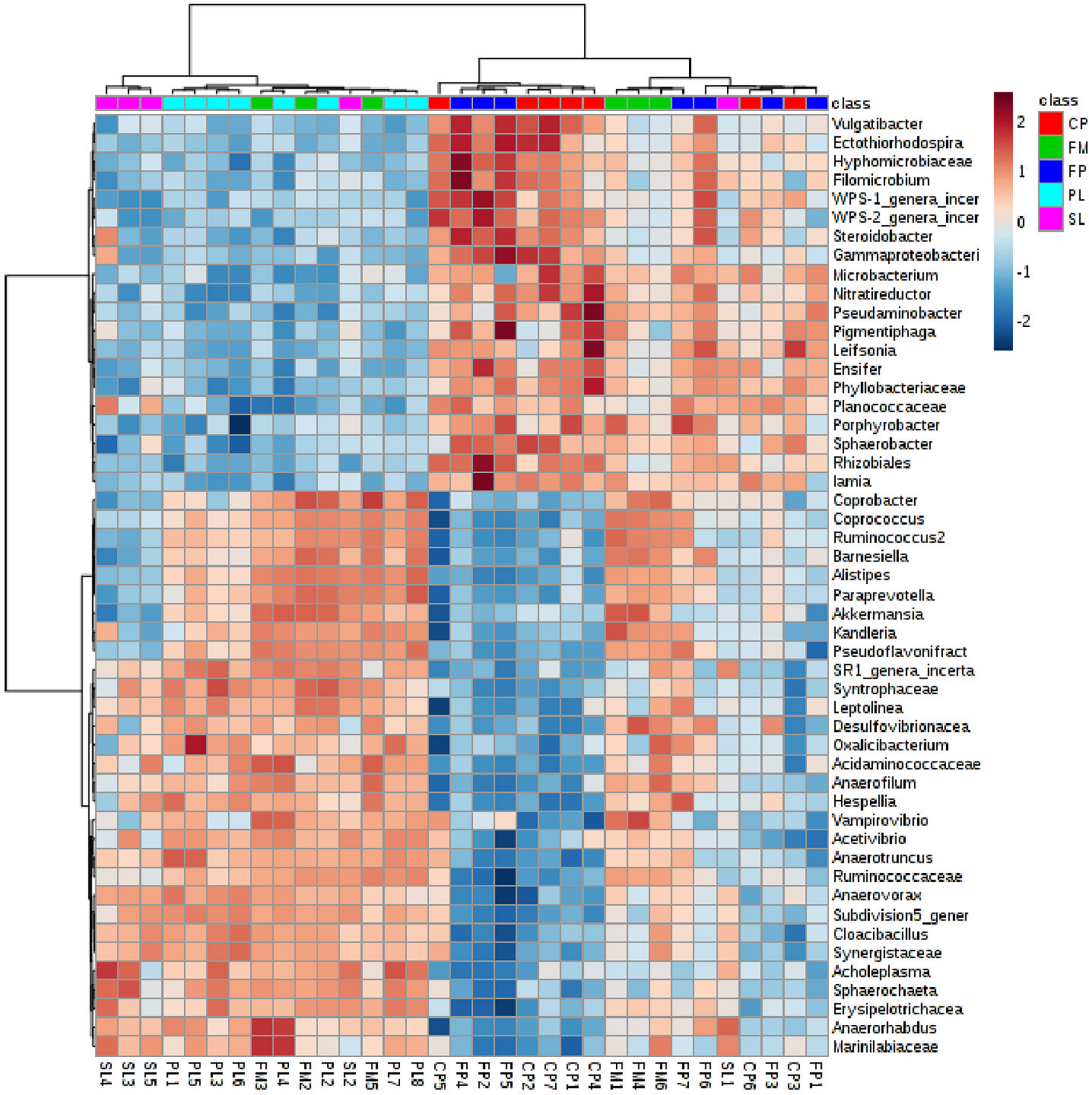
Microbial community heat map of 1818 taxa in flush manure (FP), Primary lagoon manure (PL), Secondary lagoon manure (SL), Fresh pile (FP) and Compost/dry pile (CP). At the top of heat map, dark red color indicates CP, green color indicates FM, blue indicates FP, aqua color indicates PL, and pink color indicates SL.

FP samples, which were not dried and composted, showed the abundance of bacteria of genus *Acinetobacter* and *Enterococcus*; these bacteria were the most common and accounted for 3.5% - 39.53% and 4.8% - 11.86%, respectively. In CP samples, which were either dried or composted, the proportion of bacteria of genus *Acinetobacter* ranged from 18.3% to 19.2%. Other abundant species in CP samples were *Flavobacteriaceae*, *Bacillaceae*, *Pseudoxanthomonas*, *Clostridia*, and *Sphingobacterium*, accounting for 3.9–24.9%, 7.2–7.3%, 2.8–5.5%, 4.5–6%, 2.1–3.1%, respectively. Other abundant species in FP samples were *Bacteriodetes*, *Trichococcus*, *Clostridiales*, *Flavobacterium*, and *Psychrobacter*. A heat map of the top 50 taxa in total of 1818 taxa is shown in Fig 3. In FM samples, the most common species were *Ruminococcaceae* varying from 7.2 to 13.1%. Species such as *Bacteroidetes* and *Clostridium* varied 4.2–11.5% and 3.5–9.7%. In lagoon samples (PL and SL), however, the most common species were *Bacteroidetes*, *Flavobacteriaceae*, and *Psychrobacter* accounting for 11.1–15.9%, 3.3–13.1%, and 19.3–29.6%, respectively. Dendrograms and PCA plots are shown in supplementary figures (S1 Fig).

**Fig 4:**
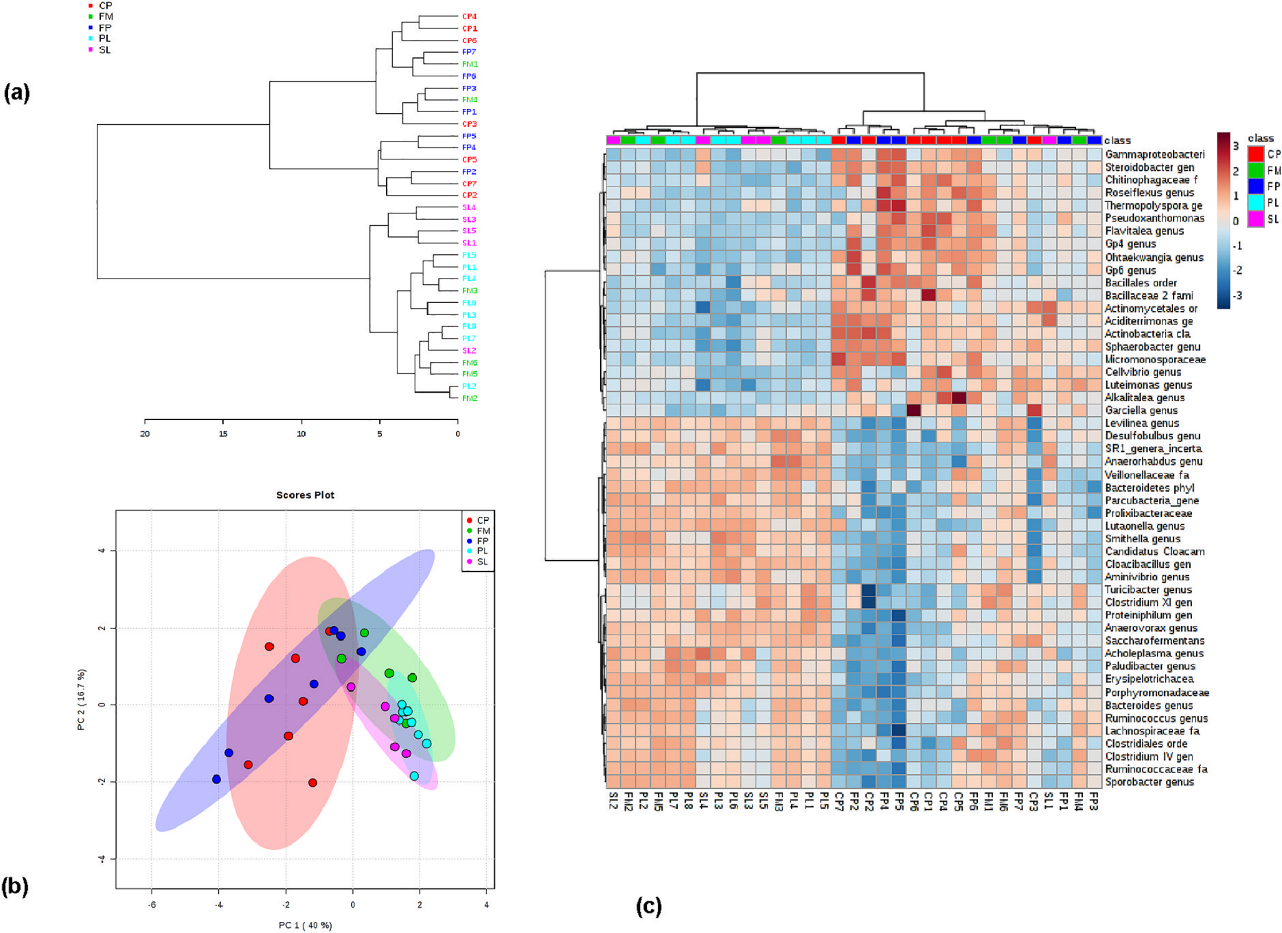
Microbial community diversity in CP, FP, FM, PL, and SL samples. a) dendrogram plot; b) PCA plot; c) heat map of CP, FP, FM, PL, and SL.

The application of algorithm based on the abundance criteria (organisms ≥ 1% in at least 2 samples, or organisms ≥ 5% in at least 1 sample) resulted in 128 taxa, and the analysis of top 50 communities in 128 taxa is shown in Fig 4. The dendrogram and PCA of CP, FM, FP, PL, and SL are shown in Fig 4A and 4B, respectively. The heat map (Fig 4C) shows the distribution of microbial communities in CP, FM, FP, PL, and SL. In the dendrograms, the horizontal axis represents the distance of dissimilarity between clusters. The vertical axis represents objects and clusters. Results showed that FM is more similar to PL than SL. Furthermore, the similarity between FM and PL was greater than SL and CP. The frequency and abundance in 128 taxa of solid manure samples are shown in supplementary file (S1 Table), and these characteristics for liquid samples are shown in supplementary table (S2 Table).

Sample grouping tendency in 128 taxa was evaluated using PCA. The PCA score plot (Fig 4B) shows a two-dimensional plot of 33 samples. The first two principal components (PC) explained 56.7% (40% + 16.7%) of the total variance in the microbial community composition. FP and CP samples were clustered together mostly in the upper and lower left corner of the plot, while FM, PL, and SL were clustered together in the lower left of the plot. As shown in the figure, CP and FP groups were similar to each other and distinct from PL, SL, and FM. Further, PL and SL were grouped together. The clear separation of CP and FP (solid manure) from PL, SL, and FM (liquid manure) indicates that manure handling processes such as solid-liquid separation adapted in dairy farms have the potential to alter the microbial communities in the manure. Together, these results demonstrate that the forms of manure fertilizers (i.e., liquid and solid) will affect the microbial quality of manure fertilizers, which prove the central idea of our hypothesis.

The abundance of genera in each sample is shown in a heat map (Fig 4C). In the heat map, the light blue indicates low abundance and dark red indicates high abundance. The results of top 50 genera present in samples indicate that species abundance differs among samples. In FP, the most abundant species such as *Acinetobacter*, *Psychrobacter*, and *Enterococcus* accounted for 9.9%, 2.5%, and 2.5%, respectively. The other unclassified bacteria in FP accounted for 31%. In CP, the most abundant species include *Planifilum*, *Acinetobacter*, and *Flavobacteriaceae* accounted for 6.4%, 6.0%, and 4.4%, respectively. The unclassified bacteria in CP were 25.6%.

### 3.2 Comparison of microbial communities in solid versus liquid samples

**Fig 5:**
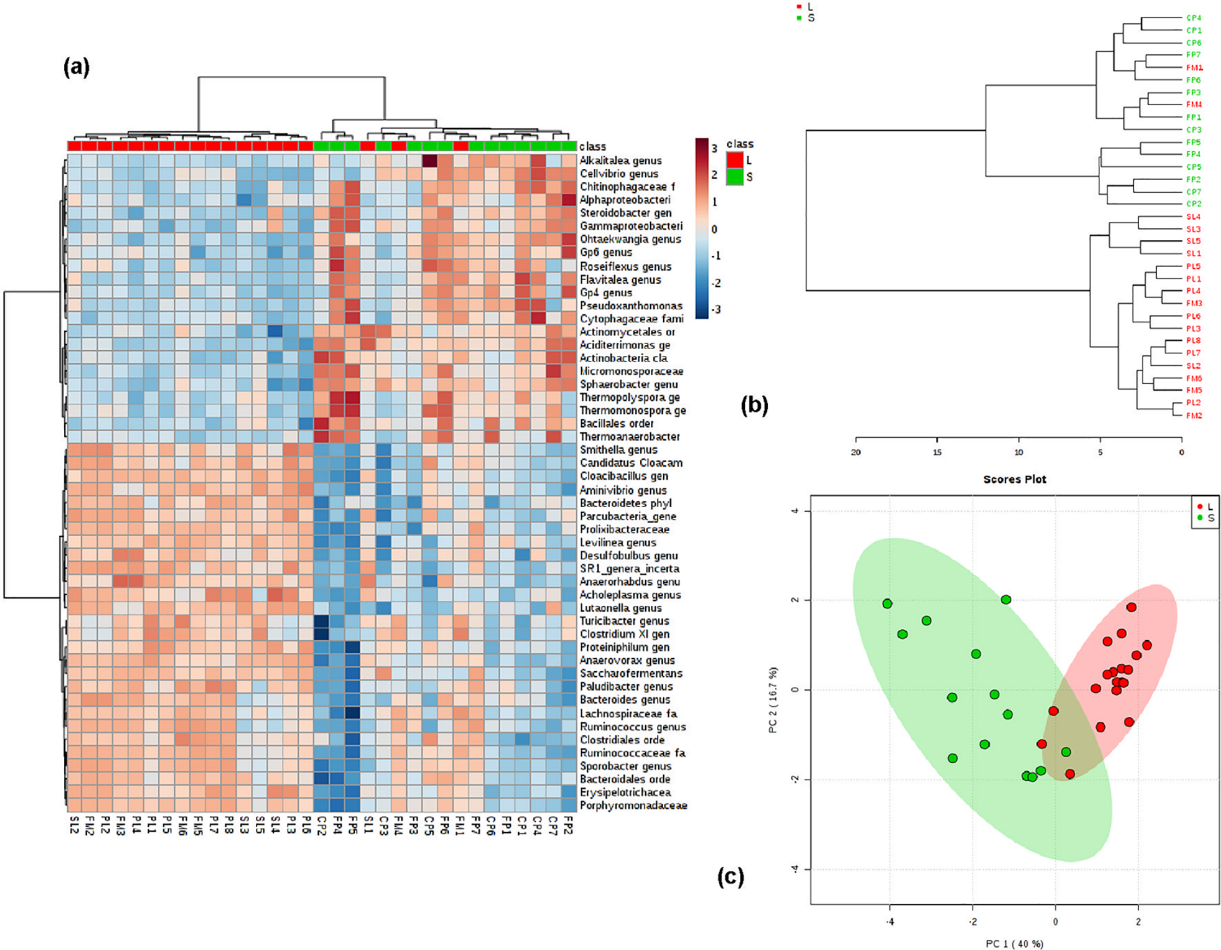
The distinction of microbial community in solid and liquid manure samples. a) heat map of liquid and solid manure samples; b) dendrogram plot; and c) PCA plot of solid and liquid manure samples.

To understand the distinction among microbial communities in solid and liquid samples, the data of liquid samples (PL, SL, FP) and solid samples (CP and FP) were grouped separately. The dendrogram plot showed clustering among solid and liquid samples (Fig 5A). Results indicated that all the liquid samples were more similar to each other than solid samples. The PCA plot (Fig 5B) displayed that the solid samples were mostly clustered to the left side, while the liquid samples were clustered to the right side, indicating a clear separation among liquid and solid samples. The top 50 genera in 128 taxa are presented in heat map (Fig 5C). Results showed that the top 22 species listed at the top of heat map were more abundant in solid samples compared to the liquid. These species include *Bacteroidetes* (8.4%), *Ruminococcaceae* (6.4%), *Flavobacteriaceae* (6.0%), *Clostridium* (4.4%), *Cloacibacillus* (3.4%), *Petrimonas* (3.2%), *Psychrobacter* (2.9%), and *Proteiniphilum* (1.8%). The bottom 28 species were more abundant in liquid samples compared to solid samples, and these microbial communities include *Smithella* (0.9%), *Pseudomonas* (0.8%), *Sporobacter* (0.7%), *Treponema* (0.6%), and *Aminivibrio* (0.5%).

**Fig 6:**
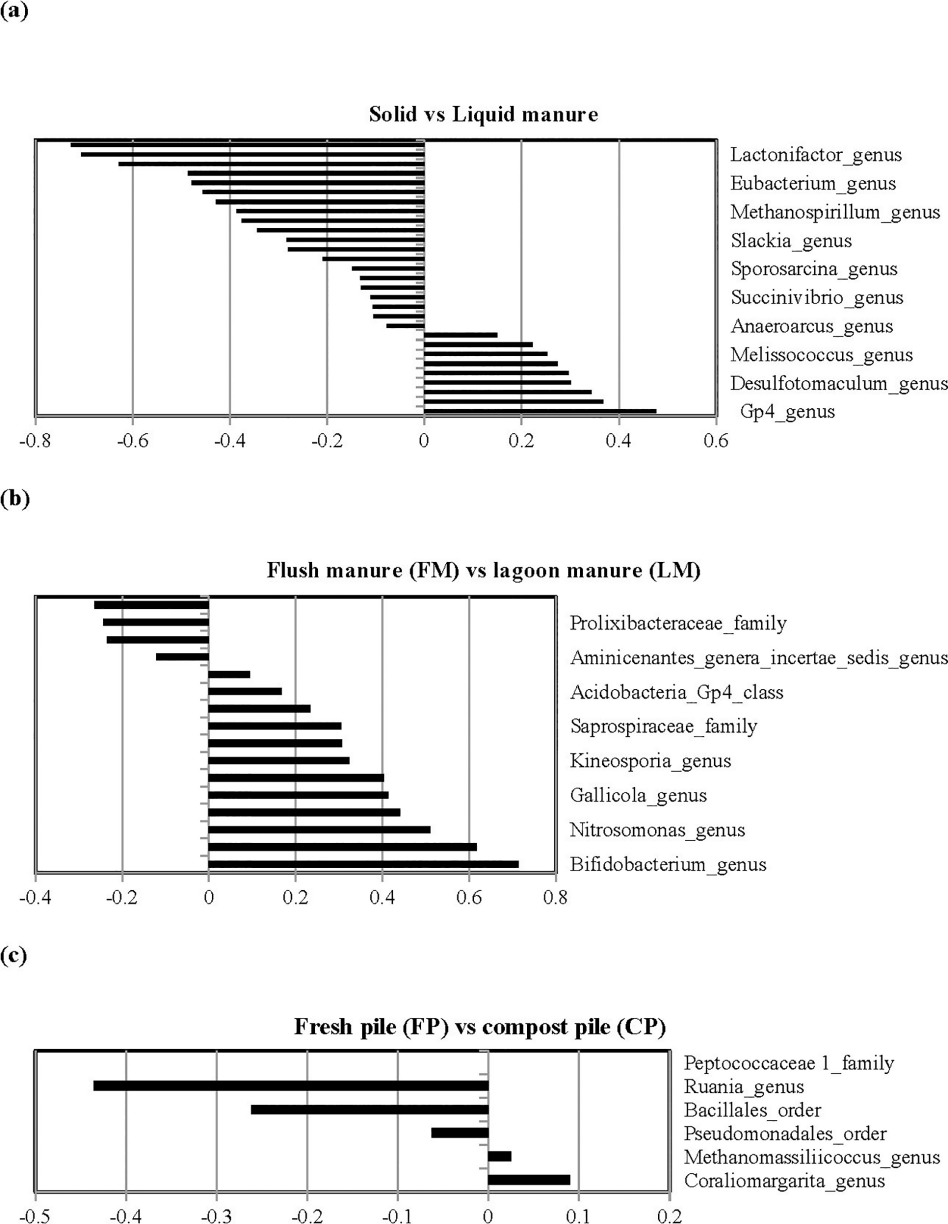
Canonical structure coefficients. a) correlation between each microbial taxa and the discriminant function for solid vs liquid manure. Bacteria with canonical structure coefficients of -0.3 or +0.3 are considered important when differentiating solid and liquid manure; b) correlation between each microbial taxa and the discriminant function for FM vs LM; and c) correlation between FP and CP.

Based on the canonical analysis (Fig 6), the genus *Gp4*, *Nocardioides* and *Caryophanon* were highly correlated with solid manure, while the genus *Succiniclasticum*, *Porphyromonas*, *Methanospirillum*, *Anaeroplasma*, *Armatimonadetes*, *Eubacterium*, *Vampirovibrio*, *Anaerovorax* and *Lactonifactor*, and the family *Porphyromonadaceae* were highly correlated with liquid manure (Fig 6). Canonical values from the discriminate analysis were also used to identify bacteria that were highly correlated and led to differentiation of FP vs CP, and FM vs LM (PL and SL combined). Bacteria genus *Coraliomargarita* was highly correlated with FP and genus *Ruania* and family *Peptococcaceae* were highly correlated with CP. From this analysis, we observed that the genus *Bifidobacterium*, *Murdochiella*, *Nitrosomonas*, *Arcanobacterium*, *Gallicola*, and *Kurthia* were highly correlated with FM. Overall, the comparison of FM and LM had a more similar microbial composition and diversity than the comparison of FP and CP.

## 4. Discussion

Previous studies emphasized that microbiota change depending on whether it is associated with solid particles or liquid fractions [42, 43, 44]. As a consequence, the mode of manure application (i.e., liquid or solid) will likely to influence the microbial load in the cropland receiving manure as fertilizer. Both liquid and solid manure are applied as fertilizer in developing as well as developed countries; however, a detailed research in terms of microbial communities of compost manure (solid) and irrigation manure (liquid) is rare—if not unavailable. Increasing public concern with regards to the microbial load in manure fertilizers and associated health risks necessitates the scope of such studies.

Moreover, the trend in dairy industry shows that larger dairies, which confine the relatively large animal population in limited acreages, are more efficient than smaller dairies, and their number is increasing consistently. This means that the manure production in future dairy farms will increase as a result of higher animal density in a relatively limited area. Eventually, the increased manure production will be applied in cropland with or without treatment. There are many treatment options for manure, including anaerobic digestion, composting, lagoon, and drying, and the impacts of these methods on microbial population are relatively unknown at a large scale.

In addition to manure treatment methods, both environmental and dairy farm specific factors influence the microbial communities in manure [42, 43, 45, 46, 47, 48]. Previous studies, which have explored the microbial community in anaerobic digestion treatment of various waste including sludge, dairy manure, and slaughterhouse waste, indicated the presence of microbial communities of *Bacteroidetes*, *Proteobacteria*, *Firmicutes*, *Chloroflexi*, *Spirochetes*, *Clostridia*, and *Synergistia* [48, 49, 49, 50]. Other studies reporting the microbial population of cow gut indicated the presence of various microbial communities including *Spirochaetes*, *Flavobacteria*, *Sphingobacteria*, *Actinobacteria*, *Chloroflexi*, *Firmicutes*, and *Proteobacteria* species. Of these, all studies dealing with animal waste-borne microbial pathogens indicated that animal waste may act as a reservoir of human pathogens, and it has a potential to contaminate ambient water resources and pose risk to public and animal health.

The risk of microbial pollution caused by the application of manure fertilizer can be minimized by improving the existing understanding of microbial population in manure, and the effects of available treatment methods, which are in general used or recommended. This reconnaissance research based on our hypothesis proved that a relatively large microbial population persists in manure even after treatment. Regardless of composting, drying, solid-liquid separation, and lagoon, a diverse microbial population that includes pathogenic bacteria resides in manure, and the elimination of these microbial pathogens in manure requires further research. The ranking of top 15 species in FP and CP (solid samples) is shown in Table 1. In general, the abundance of bacteria for FP and CP was different than the abundance in FM, PL, and SL (Fig 4C). As an example, the top right corner showed the high abundance of microbial communities mostly in CP and FP, and these microbial communities were less abundant in top left corner of heat map mostly showing FM, PL, and SL (liquid samples). Similarly, species such as *Desulfobulbus*, *Bacteroidetes*, *Clostridiales*, *Clostridium*, and *Ruminococcaceae* were more abundant in FM, PL, and SL than in CP and FP (Fig 4C). A heat map displaying the bacterial community in liquid samples (FM, PL, and SL) and solid samples (FP and CP) and corresponding PCA plots are shown in supplementary figures (S2 Fig).

**Table 1:** Family and genera in solid dairy manure sample [Fresh pile (FP) and compost (dry) pile (CP)].

	Fresh pile (FP)				Compost manure pile (CP)			
SN	Name	Class	Avg.	±Std.	Name	Class	Avg.	±Std.
1	Other Bacteria	*b*	0.311	(±0.127)	Other Bacteria	*b*	0.276	(±0.087)
2	*Acinetobacter*	*g*	0.099	(±0.145)	*Planifilum*	*g*	0.064	(±0.152)
3	*Psychrobacter s*	*g*	0.025	(±0.043)	*Acinetobacter*	*g*	0.060	(±0.088)
4	*Saccharibacteria*	*g*	0.020	(±0.033)	*Flavobacteriaceae*	*f*	0.044	(±0.091)
5	*Roseiflexus*	*g*	0.015	(±0.034)	*Tepidimicrobium*	*g*	0.031	(±0.080)
6	*Trichococcus*	*g*	0.014	(±0.018)	*Corynebacterium*	*g*	0.018	(±0.042)
7	*Anaerolineaceae*	*f*	0.014	(±0.025)	*Pseudoxanthomonas*	*g*	0.016	(±0.025)
8	*Pseudoxanthomonas*	*g*	0.013	(±0.026)	*Alkalitalea*	*g*	0.014	(±0.034)
9	*Flavobacteriaceae*	*f*	0.012	(±0.006)	*Bacillaceae*	*f*	0.013	(±0.027)
10	*Luteimonas*	*g*	0.011	(±0.008)	*Flavobacterium*	*g*	0.011	(±0.014)
11	*Steroidobacter*	*g*	0.010	(±0.012)	*Bacillus*	*g*	0.010	(±0.019)
12	*Flavobacterium*	*g*	0.010	(±0.009)	*Luteimonas*	*g*	0.010	(±0.009)
13	*Ruminococcaceae*	*f*	0.010	(±0.014)	*Rhodospirillaceae*	*f*	0.009	(±0.012)
14	*Corynebacterium*	*g*	0.010	(±0.013)	*Petrimonas*	*g*	0.008	(±0.013)
15	*Facklamia*	*g*	0.010	(±0.012)	*Pseudomonas*	*g*	0.008	(±0.011)

Considering that manure is abundantly used as fertilizer [51, 52, 53], we hypothesized that the methods of manure handling may have different impacts on microbial population in liquid manure. We examined the top 15 microbial communities in liquid manure samples obtained from lagoons. Table 2 indicates the rankings of top 15 microbial communities in FM, PL, and SL samples. In PL, *Bacteroidetes*, *Ruminococcaceae*, and *Cloacibacillus* accounted 10.6%, 6.7%, and 4.5%, respectively. The unclassified bacteria in PL accounted 12.4%. Compared to PL, the three most abundant species in FM were *Ruminococcaceae*, *Clostridium*, and *Flavobacteriaceae* accounting for 8.9%, 5.1%, and 2.8%, respectively. The unaccounted bacteria in FM were 18.1%. The abundance of the top three species (*Psychrobacter*, *Flavobacteriaceae*, and *Cloacibacillus*) in SL samples was 9.8%, 7.6% and 2.6%, respectively. Moreover, the pathogenic bacteria of genus *Clostridium* persist in all three types of liquid samples (FP, PL, and SL). Compared to liquid manure samples, this population was not as dominant in solid manure samples. Solid manure, which was collected in this study, had been passed through either a compost or piling system. One plausible reason could be ascribed to the elevated temperature of manure piles. In general, the temperature profile of compost piles reaches to 55–60°C, while the temperature of lagoon manure remains low (25–30°C). Considering our sampling strategy, which involves collecting samples from multiple dairies, certain differences in microbiota among solid and liquid samples are expected, and results are tabulated in Tables 1 and 2. The ranking of top 15 species in overall solid and liquid samples was developed, and results are shown in Table 3. The common species (of those top 15 species) in solid and liquid samples include *Flavobacteriaceae*, *Ruminococcaceae*, and *Pseudomonas*.

**Table 2:** Family and genera abundance in liquid manure samples (flushed, primary lagoon, secondary lagoon manure).

SN	Flushed manure (FP)			Primary lagoon manure (PL)			Secondary lagoon manure (SL)		
	Name	Class	Avg (± std.)	Name	Class	Avg (± std.)	Name	Class	Avg (± std.)
1	Other Bacteria	*b*	0.181	Other Bacteria	*b*	0.124	Other Bacteria	*b*	0.162
			(±0.033)			(±0.026)			(±0.082)
2	*Ruminococcaceae*	*f*	0.09	*Flavobacteriaceae*	*f*	0.073	*Psychrobacter*	*g*	0.098 (±0.138)
			(±0.024)			(±0.036)			
3	*Clostridium XI*	*g*	0.051	*Ruminococcaceae*	*f*	0.067	*Flavobacteriaceae*	*f*	0.076
			(±0.030)			(±0.038)			(±0.055)
4	*Flavobacteriaceae*	*f*	0.028	*Petrimonas*	*g*	0.051	*Clostridium XI*	*g*	0.03
			(±0.021)			(±0.068)			(±0.039)
5	*Cloacibacillus*	*g*	0.025	*Clostridium XI*	*g*	0.048	*Cloacibacillus*	*g*	0.026
			(±0.020)			(±0.051)			(±0.023)
6	*Trichococcus*	*g*	0.024	*Cloacibacillus*	*g*	0.045	*Ruminococcaceae*	*f*	0.026
			(±0.017)			(±0.037)			(±0.035)
7	*Facklamia*	*g*	0.024	*Proteiniphilum*	*g*	0.026	*Pseudomonas*	*g*	0.025
			(±0.027)			(±0.022)			(±0.044)
8	*Lachnospiraceae*	*f*	0.024	*Anaerovorax*	*g*	0.02	*Petrimonas*	*g*	0.021
			(±0.009)			(±0.015)			(±0.016)
9	*Saccharibacteria*	*g*	0.024	*Bacteroides*	*g*	0.017	*Planomicrobium*	*g*	0.019
			(±0.021)			(±0.012)			(±0.034)
10	*Clostridium IV*	*g*	0.017	*Candidatus Cloacamonas*	*g*	0.017	*Acholeplasma*	*g*	0.018
			(±0.009)			(±0.019)			(±0.019)
11	*Bacteroides*	*g*	0.016	*Lutaonella*	*g*	0.014	*Proteiniphilum*	*g*	0.017
			(±0.015)			(±0.007)			(±0.014)
12	*Turicibacter*	*g*	0.014	*Turicibacter*	*g*	0.011	*Candidatus cloacamonas*	*g*	0.016
			(±0.009)			(±0.010)			(±0.019)
13	*Psychrobacter*	*g*	0.014	*Veillonellaceae*	*f*	0.011	*Chromatiaceae*	*f*	0.014
			(±0.027)			(±0.009)			(±0.028)
14	*Petrimonas*	*g*	0.012	*Clostridium XlVa*	*g*	0.01	*Veillonellaceae*	*f*	0.012
			(±0.011)			(±0.004)			(±0.004)
15	*Porphyromonadaceae*	*f*	0.011	*Levilinea*	*g*	0.009	*Anaerovorax*	*g*	0.012
			(±0.005)			(±0.005)			(±0.004)

**Table 3:** The ranking of microbial community abundance in overall solid and liquid dairy manure samples.

	Solid (S)			Liquid (L)		
SN	Name	Class	Avg.	Name	Class	Avg.
1	*Other Bacteria*	*b*	0.293	Other Bacteria	*b*	0.150
2	*Acinetobacter*	*g*	0.079	*Ruminococcaceae*	*f*	0.064
3	*Flavobacteriaceae*	*f*	0.028	*Flavobacteriaceae*	*f*	0.060
4	*Psychrobacter*	*g*	0.015	*Clostridium*	*g*	0.044
5	*Pseudoxanthomonas*	*g*	0.015	*Cloacibacillus*	*g*	0.034
6	*Corynebacterium*	*g*	0.014	*Petrimonas*	*g*	0.032
7	*Saccharibacteria*	*g*	0.014	*Proteiniphilum*	*g*	0.018
8	*Flavobacterium*	*g*	0.011	*Candidatus cloacamonas*	*g*	0.015
9	*Luteimonas*	*g*	0.010	*Anaerovorax*	*g*	0.014
10	*Anaerolineaceae*	*f*	0.009	*Bacteroides*	*g*	0.013
11	*Ruminococcaceae*	*f*	0.008	*Turicibacter*	*g*	0.011
12	*Bacillus*	*g*	0.007	*Veillonellaceae*	*f*	0.010
13	*Micromonosporaceae*	*f*	0.007	*Levilinea genus*	*g*	0.008
14	*Pseudomonas*	*g*	0.007	*Clostridium*	*g*	0.008
15	*Bacillaceae*	*f*	0.007	*Pseudomonas*	*g*	0.008

As asserted in our hypothesis, the level of microbial population in manure fertilizer changes with the mode of samples (i.e., liquid and solid), which indicates that the treatment methods such as composting may have different impacts on manure in terms of microbial population compared to lagoon system. The results listed in Table 3 and Fig 5 prove our hypothesis to be true. Overall results showed that manure pile samples (solid) cluster together, while the flushed manure and lagoon samples (liquid) cluster together. Additionally, fresh solid samples cluster with the flush manure samples, indicating a certain degree of microbial commonality in untreated fresh liquid and solid samples. The distinct microbial communities in solid and liquid samples might be attributed to the varying effects of the anaerobic process in lagoon environment and composting process in the pile system. Interestingly, fresh piles and old piles did not show considerable differences in microbial communities, which suggest a need for further investigation to understand the effect of manure drying and composting on the change in microbial communities. In general, primary lagoon samples showed relatively high clustering. Secondary lagoon samples were less varied, which suggest that over time, microbial communities in lagoon environment develop similar profiles. Future studies focused on understanding the effect of manure retention time in lagoon microbial community and functional profile can provide additional insights needed for evaluating the microbiota of manure fertilizers. The results of this study suggest that the microbial diversity can potentially change during manure handling, and adapting suitable methods may influence cropland soil microbiota positively.

## 5. Conclusions

Excessive application of dairy manure as fertilizer is considered to be a cause microbial pollution in ambient water. To understand the potential impact of dairy manure application as fertilizer in terms of microbial pollution and diversity, here we studied the microbiome of dairy manure under various treatment conditions. Analysis was performed on the flushed manure, solid manure, and manure of lagoon systems. The 16S rRNA-based microbial analysis demonstrated that a large, diverse bacterial population inhabits the manure and changes with manure treatments. Results showed a considerable difference in population among microbiomes of liquid and solid manure. The microbiomes of primary and secondary lagoon manure were comparable. The microbial populations of fresh manure piles and old manure piles were similar, which might be attributable to a lesser impact of composting and drying under the studied conditions. The considerable differences among microbiomes of liquid and solid samples indicate that the application of solid manure as fertilizer may have different impacts on cropland in terms of microbial population compared to when liquid manure is applied as fertilizer.

## Supporting information

S1 Table Taxa (218) data of liquid samples. (XLSX)

S2 Table Taxa (218) data of solid samples. (XLSX)

S1 Fig Dendrogram and PCA plots for 1818 taxa of CP, FP, FM, PL, and SL samples. (TIF)

S2 Fig Heat map and PCA plot of liquid samples (FM, PL, and SL). (TIF)
