# Supplementary figures and images for: 16S rRNA analysis of diversity of manure microbial community in dairy farm environment
Source: PLoS One. 2018 Jan 5;13(1):e0190126. doi: 10.1371/journal.pone.0190126 (PMC5755784; doi:10.1371/journal.pone.0190126)

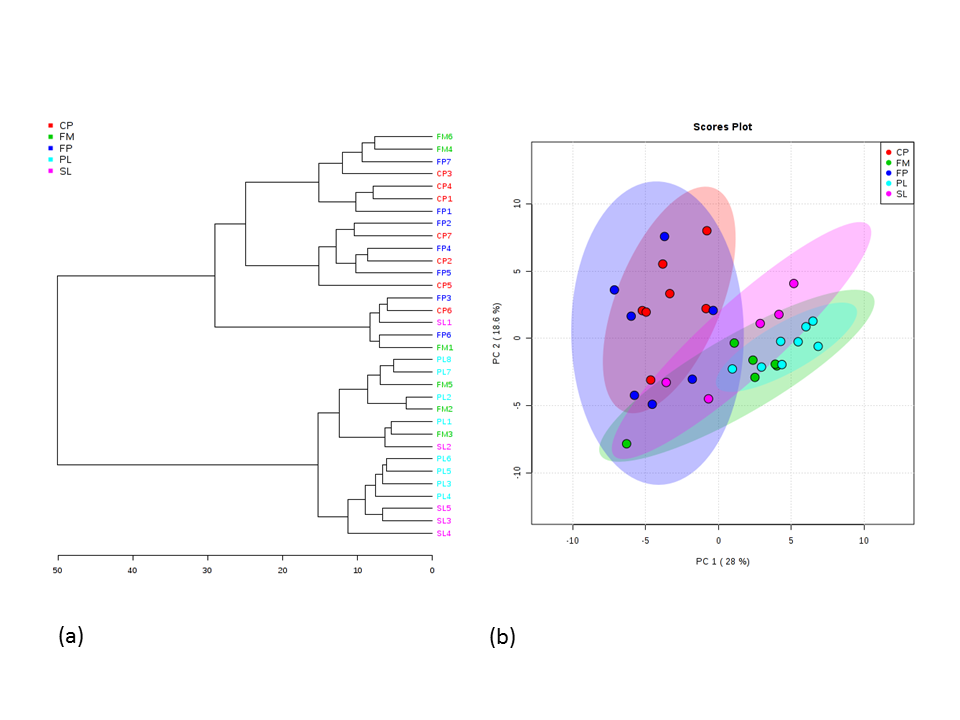

Supplement: S1 Fig — (TIF) [file pone.0190126.s003.tif]

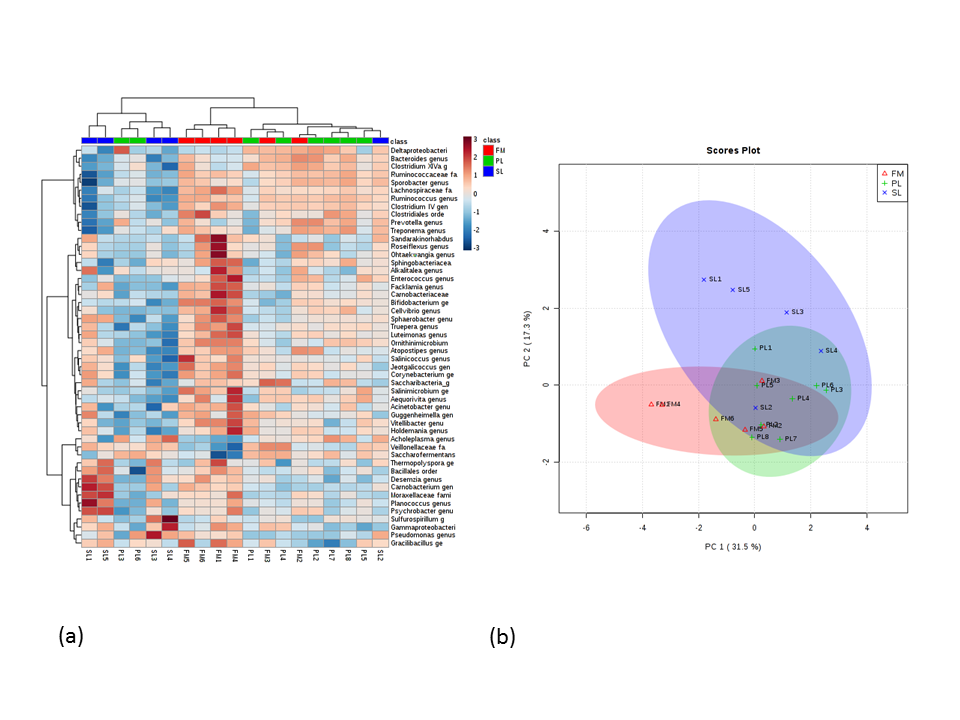

Supplement: S2 Fig — (TIF) [file pone.0190126.s004.tif]
